# Comparing Descriptive Statistics for Retrospective Studies From One-per-Minute and One-per-Second Data

**DOI:** 10.3389/fped.2022.845378

**Published:** 2022-05-12

**Authors:** Hylke H. Salverda, Janneke Dekker, Ruben S. G. M. Witlox, Peter A. Dargaville, Steffen Pauws, Arjan B. te Pas

**Affiliations:** ^1^Division of Neonatology, Department of Pediatrics, Leiden University Medical Center, Leiden, Netherlands; ^2^Paediatrics, Royal Hobart Hospital, Hobart, TAS, Australia; ^3^Menzies Institute for Medical Research, University of Tasmania, Hobart, TAS, Australia; ^4^Department of Communication and Cognition, Tilburg Center for Cognition and Communication, Tilburg School of Humanities and Digital Sciences, Tilburg University, Tilburg, Netherlands

**Keywords:** neonatology, technology, data, methodology, retrospective

## Abstract

**Background:**

Large amounts of data are collected in neonatal intensive care units, which could be used for research. It is unclear whether these data, usually sampled at a lower frequency, are sufficient for retrospective studies. We investigated what to expect when using one-per-minute data for descriptive statistics.

**Methods:**

One-per-second inspiratory oxygen and saturation were processed to one-per-minute data and compared, on average, standard deviation, target range time, hypoxia, days of supplemental oxygen, and missing signal.

**Results:**

Outcomes calculated from data recordings (one-per-minute = 92, one-per-second = 92) showed very little to no difference. Sub analyses of recordings under 100 and 200 h showed no difference.

**Conclusion:**

In our study, descriptive statistics of one-per-minute data were comparable to one-per-second and could be used for retrospective analyses. Comparable routinely collected one-per-minute data could be used to develop algorithms or find associations, retrospectively.

## Introduction

The wealth of routinely collected data in Neonatal Intensive Care Units (NICUs) has great potential. Morbidities, such as bronchopulmonary dysplasia, retinopathy of prematurity, and sepsis, can possibly be predicted when coupling analyses of routinely measured vital signs or derivatives with outcomes. One example is the HeRO symphony system predicting sepsis from variability of heart rate. (1) In real time, algorithms can summarize relevant data, detect anomalies, and notify bedside staff of risk factors in certain diseases. Routinely collected data could be used to develop algorithms or find associations, retrospectively.

However, it is unclear at what frequency data should be sampled. In our NICU, data are often sampled at least once per second (one-per-second data, i.e., 1 heart rate value per second) for prospective studies, but routinely collected vital parameters are only sampled once per minute (one-per-minute data). This keeps up performance of the clinical patient data management system, and prevents high costs associated with storage of data. Other NICUs may have similar infrastructure in place with data already collected and available. Although the data could be collected at a higher frequency, it is unclear whether lower frequency data are already enough.

We hypothesized that lower frequency data could, in some cases, be sufficient to run retrospective studies. In this short report, we investigated what to expect when using one-per-minute data abstracted from one-per-second data and investigated under what conditions one-per-minute data could be used.

## Materials and Methods

Routinely collected data from a previous study were used; the ethical review committee of Leiden Den Haag Delft provided a statement of no objection for obtaining and publishing the anonymized data (G19.075). (2) Data recordings were included from infants born under 30 weeks of gestation in our tertiary-level perinatal center between 1 November 2018 and 15 March 2020. Recordings were excluded if they contained no data on peripherally measured oxygen saturation (SpO_2_).

### Data Collection and Outcome Measures

Parameters collected were 2–4s averaged SpO_2_ measured by a weight-appropriate pulse-oximeter probe (LNCS Neo Masimo SET; Masimo Irvine, CA, United States), and measured inspiratory fraction of oxygen (FiO_2_). These data were sent from an SLE6000 respirator (SLE Limited, South Croydon, United Kingdom) with OxyGenie automated oxygen titration to an MP70 bedside monitor (Philips, Eindhoven, the Netherlands) or, if no respiratory support was given, SpO_2_ was measured by a Masimo module on the Philips monitor.

From the bedside monitor, data are sent to two databases: a Philips Datawarehouse Connect feed to a database in which numerical data are stored once per second for 1 year; and a one-per-minute feed (HL7 data transfer protocol), which sends the exact value at the set interval time, which may be between 5 and 60 s (in our situation, 1 per minute). The HL7 message is picked up by our patient data management system (PDMS Metavision; IMDsoft, Tel Aviv, Israel). These data are stored for at least 15 years. No filtering, anti-aliasing, averaging or other processing is done on data prior to entry in the database.

To prevent synchronization issues caused by systems running on different time clocks, we chose to process the one-per-second data into one-per-minute data: one value per minute was extracted from one-per-second data by taking the value at the change of the minute (i.e., at 0 s). For both the one-per-second and one-per-minute data for SpO_2_, we calculated the average, standard deviation, proportion of time within the target range or hypoxia (SpO_2_<80%). Within the target range was defined as SpO_2_ between 91 and 95% irrespective of FiO_2_, or 96 and 100% when room air was being inspired. For the FiO_2_, average and oxygen days were calculated. An oxygen day was defined as at least half of the data FiO_2_ values of that day above 21%. Please note that this may not represent the true oxygen exposure, as the oxygen sensor can have a deviation of 1%. Finally, the number of data points and the difference between the first and last timepoints in each dataset were noted.

Data are presented as mean (SD) and median [IQR] with standard tests for normality. Data processing and analyses were done by custom written software in MATLAB (Matlab R2020b; The MathWorks Inc., Natick, Massachusetts, United States). No statistical hypothesis testing was done as we were not testing for a difference between treatments, but examining for comparability.

## Results

**FIGURE 1:**
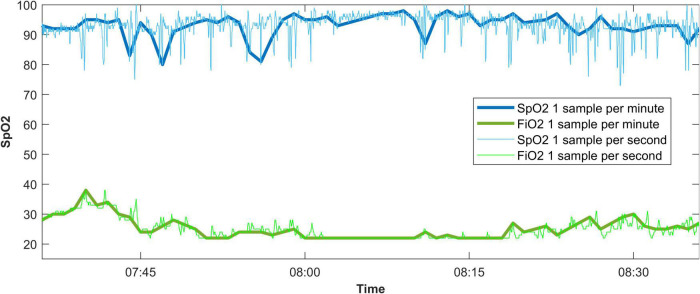
An example of a data recording displaying the effect of taking one sample every 60 s from a 1-per-second data recording. SpO_2_, oxygen saturation measured by pulse oximetry; FiO_2_, fraction of inspired oxygen.

There were data available from 92 patients, with a median of 1,151,774 [577,843–2,586,608] one-per-second data points per patient. An excerpt from a data recording is shown in Figure 1. When processed to one-per-minute data, there were 19,462 [9,129–43,162] data points left. The time difference between the first and last entries in the data recording was 375 h, 24 min, and 46 s [157:59:33–762:23:11] for the one-per-second data, and 367 h, 58 min, and 30 s [155:11:45–757:12:00] for the one-per-minute data.

In the one-per-second data, the mean SpO_2_ was 94.96 (1.88) vs. 94.96 (1.87) in the one-per-minute data (Table 1), and the standard deviation of SpO_2_ was 3.14 (0.92) vs. 3.15 (0.91), respectively. SpO_2_ was found to be within the target range in 70.96 [57.16–91.50]% of the time in the one-per-second data, and in 71.06 [57.00–91.53]% of the time in one-per-minute data. Hypoxic values under 80% were found in 0.36 [0.09–0.85]% of SpO_2_ values in the one-per-second dataset vs. 0.35 [0.10–0.85]% of SpO_2_ values for the one-per-minute dataset. Missing values were also similar [2.06 (1.59–2.91)% vs. 2.06 (1.52–2.87)%]. Bland-Altman plots can be found in the Supplementary Figures 1–6.

**TABLE 1:** Analysis of recordings.

*n* = 92		One-per-second	One-per-minute
Average SpO_2_	Mean (SD)	94.96 (1.88)	94.96 (1.87)
Standard deviation SpO_2_	Mean (SD)	3.14 (0.92)	3.15 (0.91)
Percentage SpO_2_ in target range^†^	Median [IQR]	70.96 [57.16–91.50]	71.06 [57.00–91.53]
Percentage SpO_2_ < 80%	Median [IQR]	0.36 [0.09–0.85]	0.35 [0.10–0.85]
Average FiO_2_	Median [IQR]	22.65 [21.67–24.56]	22.70 [21.69–24.59]
Days of supplemental oxygen	Median [IQR]	0 [0–2]	0 [0–2]
Percentage missing SpO_2_	Median [IQR]	2.06 [1.59–2.91]	2.06 [1.52–2.87]
Number of data points	Median [IQR]	1151774 [577843–2586608]	19462 [9129–43162]
Duration hours:min:second	Median [IQR]	375:24:46 [157:59:33–762:23:11]	367:58:30 [155:11:45–757:12:00]

*FiO_2_, fraction of inspired oxygen; SpO_2_, peripheral oxygen saturation. ^†^91 ≤ SpO_2_ ≤ 95 or SpO_2_ ≥ while FiO_2_ = 0.21.*

The per-patient-average-inspired FiO_2_ was 22.65 [21.67–24.56] vs. 22.70 [21.69–24.59], and there was no difference in oxygen days [0 (0–2) in both datasets].

Sub analyses of groups with only less than 100 h (Table 2), 200 h, and half of the total dataset showed similar results with little difference between one-per-second and one-per-minute data.

**TABLE 2:** Sub analysis of shorter recordings.

		Recordings < 100 h (*n* = 11)		Recordings < 200 h (*n* = 28)	
		One-per-second	One-per-minute	One-per-second	One-per-minute
Average SpO_2_	Mean (SD)	96.74 (1.74)	96.74 (1.67)	95.43 (2.06)	95.43 (2.04)
Standard deviation SpO_2_	Mean (SD)	3.07 (1.04)	3.17 (1.08)	3.00 (1.01)	3.03 (1.04)
Percentage SpO_2_ in target range	Median [IQR]	93.58 [52.76–98.80]	93.87 [55.43–98.85]	90.21 [60.06–96.47]	90.49 [59.62–94.69]
Percentage SpO_2_ < 80%	Median [IQR]	0.60 [0.08–0.81]	0.75 [0.14–0.96]	0.32 [0.07–0.92]	0.30 [0.07–1.01]
Average FiO_2_	Median [IQR]	21.05 [21.00–22.03]	21.05 [21.00–22.13]	21.99 [21.01–25.40]	21.95 [21.01–25.44]
Days of supplemental oxygen	Median [IQR]	0 [0–2]	0 [0–2]	0 [0–3]	0 [0–3]
Percentage missing SpO_2_	Median [IQR]	2.85 [1.43–5.16]	3.04 [1.41–5.14]	2.58 [1.65–3.45]	2.58 [1.48–3.28]
Number of data points	Median [IQR]	161742 [82864–188130]	2699 [1381–3364]	507425 [175763–542365]	8458 [3027–9041]
Duration hours:min:second	Median [IQR]	51:18:47 [32:40:20–51:17:00]	51:17:00 [24:13:00–62:04:00]	149:49:41 [55:26:45–154:20:26]	149:48:30 [55:25:45–154:20:00]

*FiO_2_, fraction of inspired oxygen; SpO_2_, peripheral oxygen. ^†^91 ≤ SpO_2_ ≤ 95 or SpO_2_ ≥ while FiO_2_ = 0.21.*

## Discussion

In this study, we found little to no difference when comparing descriptive statistics of one-per-minute data and one-per-second data from the same source. This included clinically relevant outcomes as proportion of time within the oxygen saturation target range, hypoxia, and days of supplemental oxygen. Sub analyses of recording under 100 or 200 h showed no difference. The results suggest that routinely collected data recordings of comparable length or longer could be used for retrospective studies.

Although using routinely collected vital parameters for big data analysis and machine learning is increasingly popular, to our knowledge, there is no literature available describing the minimum data sampling frequency for our purpose. From the field of data signal processing, the Nyquist-Shannon sampling theorem (3) provides us a guideline for a sufficient sample rate, but this is aimed at reproducing the original signal, and not the summarizing statistic we often require for our retrospective studies.

One could argue that taking a sample every minute from continuous vital signs monitoring is somewhat analogous to research in general. It is uneconomical to study an entire population; thus, we take a representative sample. When the change of being sampled is related to the outcome, there is a chance of biased results. Although our sample is not at random, the value is always extracted in the first second of the minute. It is unlikely that a vital parameter like the heart rate is systematically lower in the first second of the minute or, in other words, related to the outcome. There may be a detectable circadian trend in the average heart rate, but the instantaneous heart rate should not be related to a certain second within a minute.

Limitations of our study are that one-per-minute data cannot be used to calculate the length of vital sign episodes, for example, the duration of a hypoxic episode, or other more elaborate outcomes from complex signal processing techniques. We have only investigated descriptive statistics of SpO_2_ and FiO_2_, and most of the data recordings had minimum duration of 100 h. It should also be noted that intra-recording differences were present, but these are averaged out over the entire set. Finally, to prevent synchronization issues, we did not compare our PDMS data directly with our higher frequency data, but down-sampled the latter. However, because in neither case, filtering, anti-aliasing or other processing is done, they are comparable.

## Conclusion

In our study, descriptive statistics of lower frequency data were comparable to high frequency data and could be used for retrospective analyses. Comparable routinely collected one-per-minute data could be used to develop algorithms or find associations, retrospectively.

## Data Availability Statement

The raw data supporting the conclusions of this article will be made available by the authors, without undue reservation.

## Ethics Statement

The studies involving human participants were reviewed and approved by the Medical Ethical Review Committee of Leiden Den Haag Delft. Written informed consent from the participants’ legal guardian/next of kin was not required to participate in this study in accordance with the national legislation and the institutional requirements.

## Author Contributions

HS was the principal author of the study and contributed to the design of the study, performed clinical data collection, verification and analyses, and drafted the first version of the manuscript. JD and RW drafted the first version of the manuscript. PD contributed to the design of the study and drafted the first version of the manuscript. SP performed data analyses and interpretation, and drafted the first version of the manuscript. AP contributed to the design of the study and data interpretation and drafted the first version of the manuscript. All authors provided substantial intellectual contributions and approved the final version of the manuscript.

## Conflict of Interest

The authors declare that the research was conducted in the absence of any commercial or financial relationships that could be construed as a potential conflict of interest.

## Publisher’s Note

All claims expressed in this article are solely those of the authors and do not necessarily represent those of their affiliated organizations, or those of the publisher, the editors and the reviewers. Any product that may be evaluated in this article, or claim that may be made by its manufacturer, is not guaranteed or endorsed by the publisher.
