# Supplementary material for: Comparing Descriptive Statistics for Retrospective Studies From One-per-Minute and One-per-Second Data
Source: Front Pediatr. 2022 May 12;10:845378. doi: 10.3389/fped.2022.845378 (PMC9133439; doi:10.3389/fped.2022.845378)
Supplement: Supplementary file 1 [file Data_Sheet_1.docx]

Supplementary Material

## Supplementary Figures


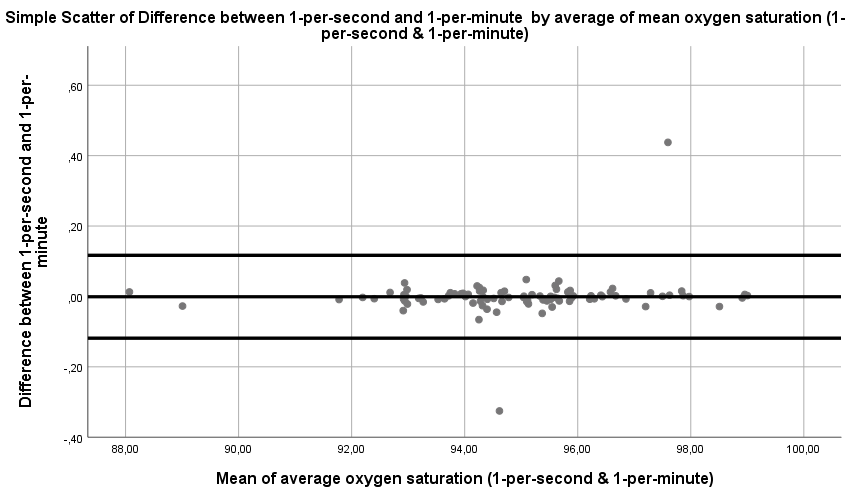


**Supplementary Figure 1.** Bland-Altman plot of average oxygen saturation 1-per-second data & 1-per-minute data


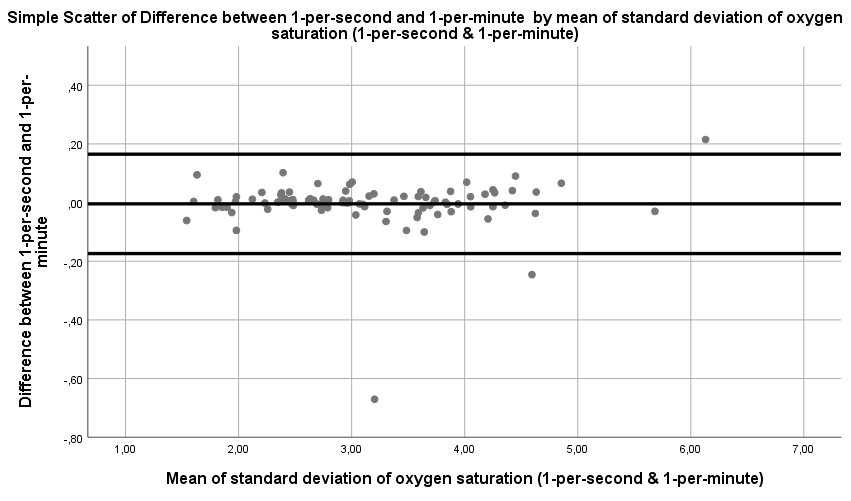


**Supplementary Figure 2.** Bland-Altman plot of standard deviation oxygen saturation 1-per-second data & 1-per-minute data


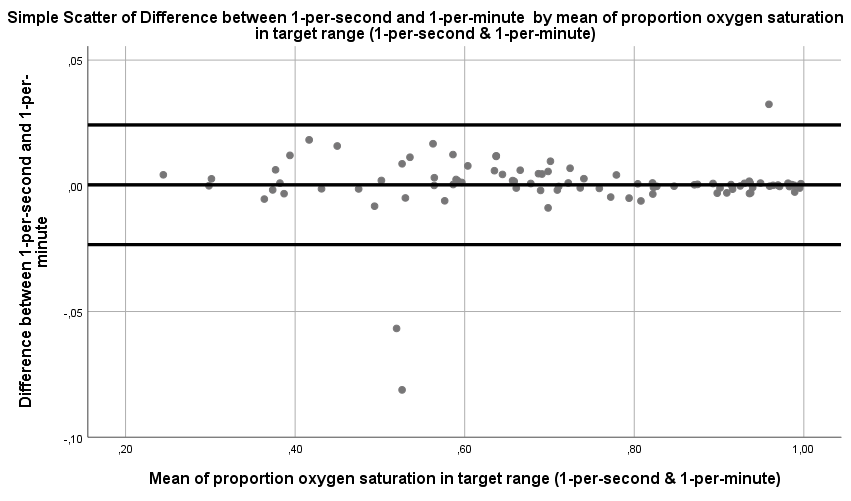


**Supplementary Figure 3.** Bland-Altman plot of oxygen saturation in target range 1-per-second data & 1-per-minute data


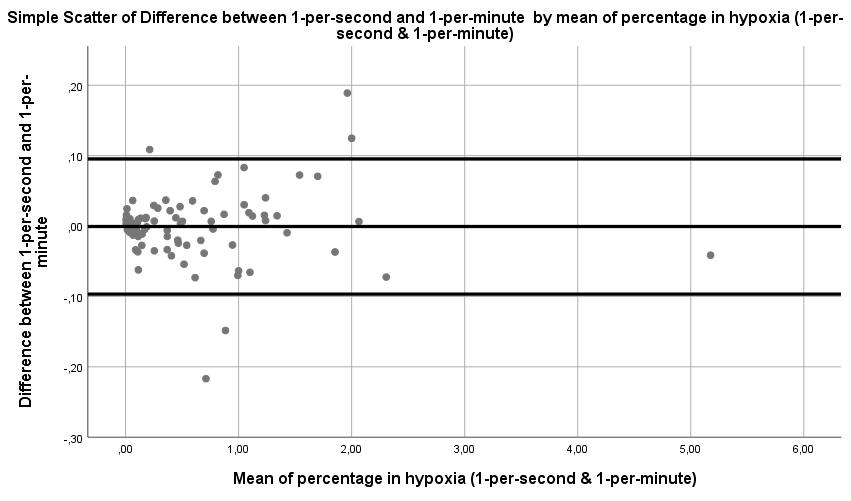


**Supplementary Figure 4.** Bland-Altman plot of hypoxia 1-per-second data & 1-per-minute data


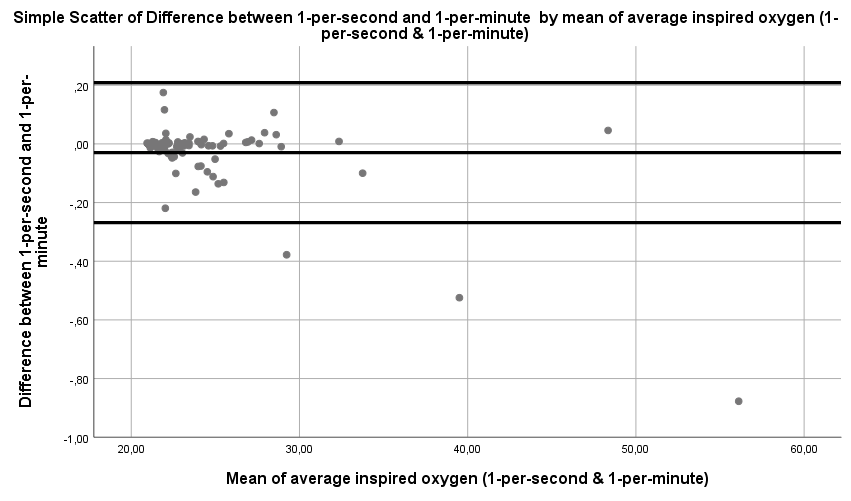


**Supplementary Figure 5.** Bland-Altman plot of average inspired oxygen 1-per-second data & 1-per-minute data


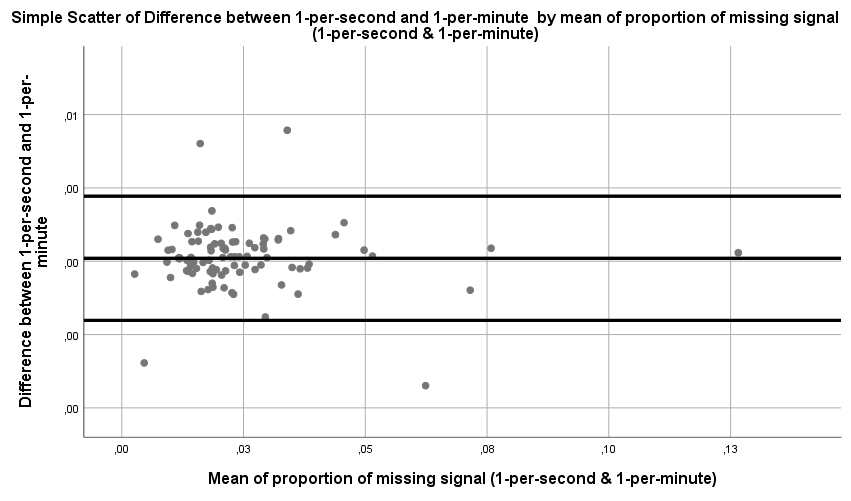


**Supplementary Figure 6.** Bland-Altman plot of missing signal 1-per-second data & 1-per-minute data
